# *Boesenbergia pandurata* Attenuates Diet-Induced Obesity by Activating AMP-Activated Protein Kinase and Regulating Lipid Metabolism

**DOI:** 10.3390/ijms13010994

**Published:** 2012-01-17

**Authors:** Dae-Young Kim, Myung-Suk Kim, Bo-Kyung Sa, Mi-Bo Kim, Jae-Kwan Hwang

**Affiliations:** 1 Department of Biotechnology, College of Life Science and Biotechnology, Yonsei University, 50 Yonsei-ro, Seodaemun-gu, Seoul 120-749, Korea; E-Mails: tadpolekim@msn.com (D.-Y.K.); g-sstainer@hanmail.net (M.-S.K.); sbk214@naver.com (B.-K.S.); 2 Department of Biomaterials Science and Engineering, Yonsei University, 50 Yonsei-ro, Seodaemun-gu, Seoul 120-749, Korea; E-Mail: mibo841120@hanmail.net

**Keywords:** *Boesenbergia pandurata* (Roxb.) Schltr., obesity, fatty liver, lipid accumulation, AMP-activated protein kinase

## Abstract

Obesity, a chronic metabolic disorder, is characterized by enlarged fat mass and dysregulation of lipid metabolism. The medicinal plant, *Boesenbergia pandurata* (Roxb.) Schltr., has been reported to possess anti-oxidative and anti-inflammatory properties; however, its anti-obesity activity is unexplored. The present study was conducted to determine whether *B. pandurata* extract (BPE), prepared from its rhizome parts, attenuated high-fat diet (HFD)-induced obesity in C57BL/6J mice. The molecular mechanism was investigated in 3T3-L1 adipocytes and HepG2 human hepatoma cells. BPE treatment decreased triglyceride accumulation in both 3T3-L1 adipocytes and HepG2 hepatocytes by activating AMP-activated protein kinase (AMPK) signaling and regulating the expression of lipid metabolism-related proteins. In the animal model, oral administration of BPE (200 mg/kg/day for 8 weeks) significantly reduced HFD-induced body weight gain without altering the amount of food intake. In addition, elevated serum levels of total cholesterol, low-density lipoprotein cholesterol, and triglycerides were suppressed by BPE administration. Fat pad masses were reduced in BPE-treated mice, as evidenced by reduced adipocyte size. Furthermore, BPE protected against the development of nonalcoholic fatty liver by decreasing hepatic triglyceride accumulation. BPE also activated AMPK signaling and altered the expression of lipid metabolism-related proteins in white adipose tissue and liver. Taken together, these findings indicate that BPE attenuates HFD-induced obesity by activating AMPK and regulating lipid metabolism, suggesting a potent anti-obesity agent.

## 1. Introduction

Obesity is a chronic metabolic disorder caused by an energy imbalance. A long-term excess of energy intake over energy expenditure results in the storage of excess energy as fat [1,2]. Obesity is characterized by enlarged fat mass and dysregulation of lipid metabolism [3]. Hyperlipidemia is caused by dysregulation of lipid metabolism, is a critical feature of obesity, and is strongly related to metabolic disorders [4]. Therefore, improving lipid metabolism is one of the most important strategies for obesity prevention and treatment [5].

Lipid metabolism is a crucial contributor to the pathogenesis of obesity [6]. Circulating free fatty acids (FFAs) are taken up by adipose, liver, and muscle tissues, where they are metabolized or stored as triglycerides (TGs) via the mitochondrial fatty acid oxidation (FAO) or lipid synthesis pathways [7]. Lipogenic proteins, including acetyl-CoA carboxylase (ACC), fatty acid synthase (FAS), stearoyl-CoA desaturase (SCD), and peroxisome proliferator-activated receptor γ (PPARγ), are expressed predominantly in adipocytes and hepatocytes. These proteins are upregulated by sterol regulatory element-binding protein 1c (SREBP-1c), which is a key transcription factor upregulating *de novo* lipogenesis [8]. The increased expression of certain lipogenic proteins significantly elevates circulating FFAs and cholesterol, which results in insulin resistance. However, proteins, such as PPARα, carnitine palmitoyl transferase 1 (CPT-1), and uncoupling proteins (UCPs), increase FAO and fatty acid clearance. In mice, downregulating lipogenic proteins and upregulating FAO proteins attenuate obesity and dyslipidemia in the high-fat diet (HFD)-induced obese rodent model [5,9–11].

AMP-activated protein kinase (AMPK) functions as a key energy sensor and is activated by energy depletion. Once activated, AMPK inactivates ACC and, therefore, increases mitochondrial FAO [12]. It has been reported that AMPK decreases fatty acid synthesis by reducing the protein level of mature SREBP-1c [13]. AMPK is also known to increase the expression of proteins involved in fatty acid β-oxidation, including PPARα, PPARγ coactivator-1α (PGC-1α), CPT-1, and UCPs [14]. Therefore, AMPK has been suggested as a therapeutic target for dyslipidemia and obesity.

Medicinal plants have been shown to prevent and treat obesity and associated metabolic disorders by regulating lipid metabolism [15]. For example, resveratrol, (-)-epigallocatechin-3-gallate, and berberine improve obesity by activating on AMPK [16–18]. *Boesenbergia pandurata* (Roxb.) Schltr. (Syn. *Kaempferia pandurata* Roxb.), which has been widely used as a medicinal plant, has been reported to possess significant anti-inflammatory and anti-oxidative properties [19,20]. In the present study, the anti-obesity effects of *B. pandurata* extract (BPE) and its molecular mechanisms were characterized *in vitro* and *in vivo*.

## 2. Results and Discussion

### 2.1. Adipogenesis, Hepatic Lipid Accumulation, and AMPK Activation

**Figure 1:**
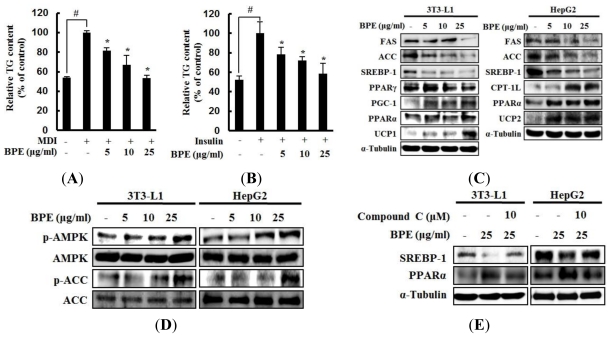
Effects of *B. pandurata* extract (BPE) on triglyceride accumulation and AMP-activated protein kinase (AMPK) activation. (**A**) Anti-adipogenic effects of BPE in 3T3-L1 adipocytes. (**B**) Inhibitory effects of BPE on hepatic lipid accumulation in insulin-induced HepG2 cells. Data are expressed as mean ± SD from three independent experiments. ^#^ *p* < 0.05 (untreated control *vs*. MDI or insulin-treated control); * p < 0.05 (BPE-treated cells *vs*. MDI or insulin-treated control). (**C**) Effects of BPE on the expression of lipid metabolism-related proteins in 3T3-L1 cells and HepG2 cells. (**D**) AMPK activation by BPE in 3T3-L1 cells and HepG2 cells. (**E**) Effects of an AMPK inhibitor, compound C, on the expression of PPARα and SREBP-1c. Each blot represents three independent experiments.

Lipid homeostasis is regulated by the fine-tuning of lipid synthesis and lipolysis, which are regulated by lipid regulatory enzymes in the peripheral tissues, such as adipose, liver, and muscle tissues [11]. To investigate the hypolipidemic effects of BPE *in vitro*, oil red O staining was performed to evaluate TG accumulation in 3T3-L1 adipocytes and in insulin-induced HepG2 hepatocytes. Treatment of 3T3-L1 adipocytes with BPE during adipogenesis markedly decreased TG accumulation (Figure 1A) without cytotoxicity (data not shown). BPE also inhibited TG accumulation in HepG2 cells stimulated by insulin (Figure 1B) without cytotoxic effects (data not shown).

To determine the mechanism by which BPE reduced TG accumulation in 3T3-L1 adipocytes and HepG2 cells, western blot analysis was performed to evaluate the expression of proteins important in lipid metabolism. BPE treatment significantly attenuated the expression of adipogenic and lipogenic proteins ACC, FAS, SREBP-1c, and PPARγ in 3T3-L1 adipocytes and HepG2 hepatocytes. This treatment also increased the expression of proteins involved in FAO (PPARα, PGC-1α, CPT-1L, and UCPs) (Figure 1C). These results suggest that BPE reduces TG accumulation in two ways: by downregulating the expression of proteins involved in lipogenesis and by upregulating proteins in the FAO pathway.

Because AMPK is a key regulator of proteins involved in lipogenesis and FAO in metabolic tissues [12], we investigated whether BPE activated AMPK by assessing its level of phosphorylation. BPE treatment drastically increased the phosphorylation of AMPK and ACC, a direct target of AMPK (Figure 1D). In addition, the effects of BPE on the expression of SREBP-1c and PPARα were reversed by compound C pretreatment, a potent AMPK inhibitor (Figure 1E). It has been reported that the phosphorylation of p38 mitogen-activated protein kinase (MAPK) by activated AMPK triggers PPARγ phosphorylation and inhibits adipogenesis [21]. BPE treatment drastically increased p38 MAPK phosphorylation (data not shown), suggesting that the inhibition of adipogenesis by BPE might be caused by PPARγ phosphorylation and reduced protein levels of mature SREBP-1c and PPARγ. These results collectively suggest that AMPK activation and regulation of lipid regulatory proteins mediate the hypolipidemic effects of BPE in 3T3-L1 adipocytes and HepG2 cells.

In our previous study, we isolated panduratin A as an active compound from BPE and evaluated its anti-obesity mechanisms in high-fat diet induced obesity [22]. Panduratin A stimulated AMPK signaling, thereby increasing lipid catabolism and decreasing lipid accumulation. LKB1 is a kinase that lies upstream of AMPK. Specifically, panduratin A activated LKB1-dependent AMPK signaling as well as change of ADP/ATP ratio, which in turn regulated PPARα/δ and lipid regulatory proteins through nuclear translocation of the AMPKα2 subunit [22]. Although BPE used in this research contained 8.0% of panduratin A as a major bioactive compound, further studies are necessary to investigate interactions among the active compounds in BPE and upstream events of AMPK.

### 2.2. Body Weight and Serum Lipid Parameters in HFD-Induced Obese Mice

**Figure 2:**
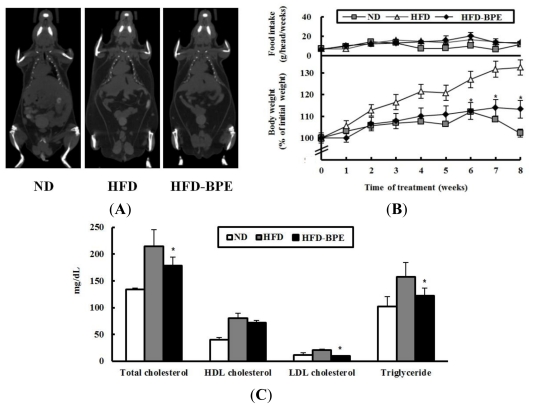
Effects of BPE on body weight and serum lipid parameters in an animal model. (**A**) Computed tomography images of the whole body of C57BL/6J mice receiving a high-fat diet (HFD) (HFD control) or a HFD with BPE (HFD-BPE). (**B**) Changes in body weight and food intake. Each point represents mean ± SD (n = 8). (**C**) Serum levels of total cholesterol, LDL cholesterol, HDL cholesterol, and triglycerides. Data are expressed as mean ± SD (n . 5). * P < 0.05 (HFD control group *vs*. HFD-BPE group).

To determine whether BPE improves HFD-induced obesity in an animal model, diet-induced obese C57BL/6J mice received oral doses of BPE (200 mg/kg/day) for 8 weeks. Micro-CT data showed that the whole body fat accumulation was lower in the HFD-BPE group compared to the HFD control group (Figure 2A). The HFD-fed mice that were treated with BPE gained about 60% less weight than the mice in the HFD control group without altering the amount of food intake (Figure 2B). These results indicate that a reduction in body weight gain by oral BPE administration is not due to a change in food intake.

Because diet-induced obesity alters serum lipids [1], we evaluated whether BPE could normalize these parameters. As shown in Figure 2C, BPE administration improved serum lipid profiles. Total cholesterol, low-density lipoprotein (LDL) cholesterol, and triglyceride levels were reduced by 19%, 55%, and 24%, respectively; however, no significant changes in the level of high-density lipoprotein (HDL) cholesterol were observed in BPE-treated mice. These data suggest that BPE might suppress obesity-induced hyperlipidemia by decreasing the serum lipid level.

### 2.3. Fat Metabolism

**Figure 3:**
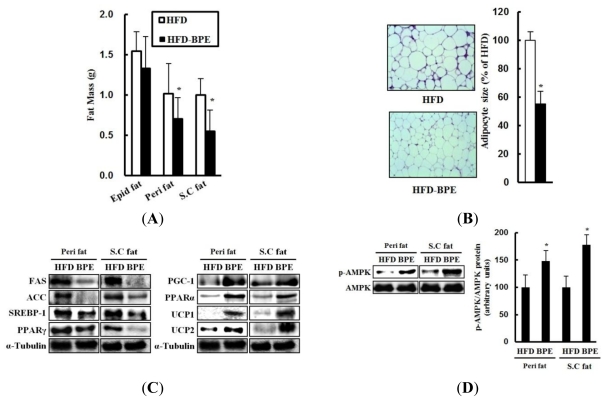
Effects of BPE on fat mass and expression of metabolic proteins in white adipose tissue (WAT). (**A**) Fat pad masses in mice fed a HFD or a HFD with BPE (HFD-BPE). Data are expressed as mean ± SD (n . 7). (**B**) Subcutaneous fat pads stained with H&E (magnification, ×200). (**C**) Metabolic protein levels in perirenal and subcutaneous fat pads. (**D**) AMPK activation by BPE in perirenal and subcutaneous fat pads. BPE denotes the HFD-BRE group. Each blot represents 8 mice in each group. * P < 0.05 (HFD control group *vs*. HFD-BPE group).

To examine whether a reduction in body weight gain by BPE was caused by a decrease in adiposity, fat pads were weighed after the mice were sacrificed. Reduced fat pad masses were observed in the epididymal (Epid) (14% less), perirenal (Peri) (25% less), and subcutaneous (S.C) (40% less) fat pads of the BPE-treated group (Figure 3A). Histological analysis of the S.C fat pad indicated that the BPE-induced reduction in fat pad mass was principally due to decreased adipocyte size (Figure 3B). These results indicate that BPE reduces fat mass by attenuating adipocyte enlargement, and this suggests an anti-adipogenic effect of BPE.

In addition to larger adipocytes, histological analysis of white adipose tissue (WAT) showed that clusters of small, nucleated cells were present in between the adipocytes from the HFD control group (Figure 3B). These clusters of nucleated cells implied macrophage infiltration into the WAT. Macrophages secrete inflammatory cytokines, such as tumor necrosis factor-α (TNF-α), causing insulin resistance in the WAT [23]. However, BPE administration decreased the clusters of these small, nucleated cells in the WAT compared to the HFD control group (Figure 3B). It has been reported that BPE has strong anti-inflammatory activity [19], suggesting that the anti-inflammatory effects of BPE might contribute to a reduced macrophage infiltration and possible treatment of dyslipidemia. However, further studies, such as macrophage-specific immunostaining and analysis of proinflammatory cytokine levels, should be conducted to determine a correlation between anti-inflammatory effects and reduced macrophage infiltration.

To determine whether the reduction in fat pad masses from the BPE-treated mice were accompanied by an altered expression of genes involved in lipid metabolism, proteins were prepared from Peri and S.C fat pads, and a western blot analysis was performed. Proteins involved in lipogenesis and adipogenesis, including ACC, FAS, SREBP-1c, and PPARγ, were reduced in these fat pads from BPE-treated mice (Figure 3C). Conversely, BPE increased the expression of lipolytic proteins, including PPARα, PGC-1α, UCP1, and UCP2, in the WAT (Figure 3C). Consistent with the *in vitro* data, we found that BPE administration activated AMPK signaling in the Peri and S.C fat pads in this animal model (Figure 3D). Taken together, these results suggest that BPE reduces fat pad mass and adipocyte size by downregulating lipogenic proteins and upregulating FAO-related proteins.

### 2.4. Hepatic Lipid Metabolism

**Figure 4:**
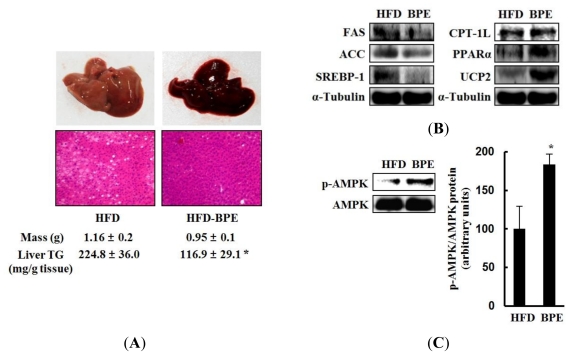
Hypolipidemic effects of BPE in the liver. (**A**) Gross appearance and weights of livers from the HFD and HFD-BPE groups. Liver tissues were stained with H&E (magnification, ×200) and hepatic triglyceride content was measured. Data are expressed as mean ± SD (n = 7). (**B**) Hepatic expression of lipid metabolism-related proteins. (**C**) AMPK activation by BPE in the liver. BPE denotes the HFD-BRE group. Each blot represents 8 mice in each group. * p < 0.05 (HFD control group *vs*. HFD-BPE group).

The development of nonalcoholic fatty liver disease is one of the most important characteristics of obesity [11]. Thus, the inhibitory effect of BPE on nonalcoholic fatty liver was evaluated by determining TG content in the livers of these animals. The gross appearance and histological analysis of the liver from the HFD group exhibited a sign of nonalcoholic fatty liver disease because many fat droplets accumulated in the liver acini; however, hepatic TG content in the HFD-BPE group was significantly decreased by 50% compared to the HFD control mice (Figure 4A). These results indicate that BPE reduces hepatic lipid accumulation and may prevent the development of a nonalcoholic fatty liver.

To understand the mechanism by which BPE decreased fat accumulation in the liver at a molecular level, western blot analysis was used to evaluate the expression of lipid metabolism-related proteins. As shown in Figure 4B, lipogenic protein levels of ACC, FAS, and SREBP-1c were significantly reduced in the liver of BPE-treated mice, consistent with the results summarized in Figure 3C. In addition, the expression of UCP2 was significantly increased by BPE treatment; however, the expression of other FAO-related proteins, such as PPARα and CPT-1L, was modestly increased in the liver of BPE-treated mice (Figure 4B). Consistent with the *in vitro* AMPK activation, BPE increased the phosphorylation of AMPK and ACC in the liver of BPE-treated mice (Figure 4C). These results suggest that BPE may activate AMPK signaling and selectively regulate the expression of lipid metabolism-related proteins, eventually leading to both the suppression of lipogenesis and the degradation of fat.

## 3. Experimental Section

### 3.1. Plant Material

Rhizomes of *B. pandurata* were collected in Jakarta, Indonesia and identified by Dr. Nam-In Baek from the Department of Oriental Medicinal Materials and Processing at Kyunghee University (Yongin, Korea). A specimen voucher has been deposited in the Department of Biotechnology at Yonsei University (Seoul, Korea). The dried *B. pandurata* rhizomes were ground and extracted with 95% ethanol, and BPE was obtained by filtration followed by solvent evaporation (yield 12.0%). BPE contained 8.0% of panduratin A as a bioactive compound [22].

### 3.2. Chemical Reagents

Insulin, 3-isobutyl-1-methylxanthine (IBMX), and dexamethasone (DEX) were purchased from Sigma (St Louis, MO, USA). Antibodies against phosphorylated ACC (Ser79), AMPK, phosphorylated AMPK (Thr172), and PPARγ were purchased from Cell Signaling Technology (Beverly, MA, USA). An antibody against α-tubulin was purchased from Calbiochem (San Diego, CA, USA). Antibodies against ACC, FAS, SREBP-1c (amino-terminal fragment), PPARα, PGC-1α, UCP1, UCP2, and CPT-1L (liver form) were purchased from Santa Cruz Biotechnology (Santa Cruz, CA, USA).

### 3.3. Cell Culture and Differentiation

3T3-L1 preadipocytes and HepG2 hepatocytes were obtained from American Type Culture Collection (ATCC; Manassas, VA, USA). They were grown in Dulbecco’s Modified Eagle’s Medium (DMEM) supplemented with penicillin (120 units/mL), streptomycin (75 μg/mL), 10% bovine calf serum (BCS; for the 3T3-L1 preadipocytes) and 10% fetal bovine serum (FBS) (Welgene; Daegu, Korea) in an atmosphere of 5% CO_2_ at 37 °C. Differentiation medium (MDI; 0.5 mM IBMX, 0.2 μM DEX, and 1.7 μM insulin in DMEM medium with 10% FBS) was added to confluent 3T3-L1 preadipocytes with BPE (5–25 μg/mL) for 8 days. The cells were then differentiated as previously described [24]. Induction of hepatic lipid accumulation in HepG2 cells was established by pretreatment with insulin (1 μM) for 12 h [25], and insulin-induced HepG2 cells were then treated with BPE (5–25 μg/mL) for another 12 h. The extent of adipogenesis and hepatic TG accumulation was determined by oil Red O staining.

### 3.4. Animal Experiments

Sixteen male C57BL/6J mice (DooYeol Biotech, Seoul, Korea) at 4 weeks of age were housed in a controlled environment (25 ± 2 °C, 55 ± 5% relative humidity, a 12 h light-dark cycle). Throughout the experiment, the mice were allowed free access to food and tap water. After acclimatization for 1 week, all mice were fed a HFD (Rodent diet D12451; Research Diets, New Brunswick, NJ, USA). This diet contained 45% of its energy from fat, 35% from carbohydrates, and 20% from protein. After 7 weeks of dietary manipulation to induce obesity, the animals were divided equally into two groups of 8. Mice in each group were either given BPE (HFD-BPE group) or vehicle alone (HFD group). BPE was dissolved in 0.5% (wt/vol) carboxymethyl-cellulose (CMC), and animals were orally administrated BPE at a dose of 200 mg/kg/day by oral sonde for 8 weeks. Mice in the HFD group were given an equal volume of CMC. Water, food intake, and body weight were measured twice per week throughout the experiment.

At the end of the 8-week, oral-administration period, all mice were sacrificed with diethyl ether after an overnight fast. Their fat pads and livers were removed, weighed, and frozen in liquid nitrogen. Micro-computed tomography (micro-CT) experiments were performed with an animal positron emission tomography (PET)/CT/single photon emission computed tomography (SPECT) system (INVEON, Siemens, Houston, TX, USA) at the Korea Basic Science Institute in Ochang. This study adhered to the Guide for the Care and Use of Laboratory Animals developed by the Institute of Laboratory Animal Resources of the National Research Council. It was approved by the Institutional Animal Care and Use Committee of Yonsei University in Seoul, Korea.

### 3.5. Histological and Biochemical Analysis

White adipose tissue (WAT) and livers were obtained from the HFD control and HFD-BPE groups and embedded in tissue-freezing medium (Leica, Wetzlar, Germany), fixed as previously described [22]. After fixation, they were stained with hematoxylin and eosin (H&E) and analyzed for hepatic lipid accumulation and adipocyte size with an Eclipse TE2000U Inverted Microscope with twin CCD cameras (magnification, ×200; Nikon, Tokyo, Japan).

Blood was collected by heart puncture from all mice and held at room temperature for 1 h. Serum was then prepared by centrifugation at 4,000 rpm for 15 min and stored at .70 °C until analysis. Serum lipid profiles were determined with a commercial enzyme-linked immunosorbent assay (ELISA) kit from R&D Systems (Minneapolis, MN, USA).

### 3.6. Measurement of Hepatic Triglyceride Content

To determine hepatic TG levels, liver homogenates were mixed with a chloroform-methanol solution (chloroform-methanol, 2:1). The mixture was inverted and shaken for 1 h and then centrifuged at 12,000 rpm for another 15 min. The bottom layer was collected and re-suspended to analyze TG content. Total lipids were measured with the enzymatic hydrolysis method.

### 3.7. Western Blot Analysis

WAT and liver specimens (approximately 100 mg each) from the HFD control and HFD-BPE groups were homogenized with lysis buffer supplemented with a protease inhibitor cocktail (Sigma). After treatment with BPE (5–25 μg/mL for 48 h), 3T3-L1 adipocytes and HepG2 hepatocytes were lysed, and whole cell lysates were collected. To measure the activation of AMPK signaling, we treated 3T3-L1 adipocytes and HepG2 cells with BPE (5–25 μg/mL) for 1 h, and whole cell lysates were collected. To evaluate the inhibitory effects of compound C on the expression of SREBP-1c and PPARα, we pretreated cells with compound C for 30 min. Protein concentrations were measured by the Bradford assay. Western blot analysis was performed according to a standard procedure using specific antibodies. Proteins were detected with the enhanced chemiluminescence (ECL) detection system (Amersham Biosciences, Little Chalfont, UK) and visualized with a LuminoImager (LAS-3000 Bio Imaging Analysis System; Fuji Film Co., Tokyo, Japan).

### 3.8. Statistical Analysis

All experiments were repeated at least three times, and each experiment was performed in triplicate. Results are presented as mean ± standard deviation (SD). Statistical analyses were performed using SPSS 9.0 (SPSS Inc., Chicago, IL, USA). Group differences were assessed by one-way analysis of variance (ANOVA) followed by a Duncan’s test. ^#^ *p* < 0.05 and * *p* < 0.05 was considered statistically significant.

## 4. Conclusions

The active constituents in *B. pandurata* rhizomes that mediate the anti-obesity effects are still unknown. It has been reported that various active compounds, such as flavones and cyclohexenyl chalcone derivatives, are present in *B. pandurata* rhizomes, and these compounds represent anti-oxidative and anti-inflammatory activities [20,26]. We previously reported that panduratin A, a major active constituent in *B. pandurata* rhizomes, shows strong anti-oxidative and anti-inflammatory activities [19,20]. Thus, studies evaluating the metabolic effects and molecular mechanisms of panduratin A on obesity are under investigation.

The present study is the first to demonstrate that BPE administration blocks HFD-induced weight gain, normalizes serum lipid parameters, and attenuates excessive lipid accumulation in the WAT and liver of mice by activating AMPK and regulating the expression of proteins involved in lipid metabolism. Although the molecular mechanisms by which BPE exerts its anti-obesity effects remain to be elucidated, BPE shows potential as a novel, natural agent for the prevention and treatment of obesity and associated fatty liver disease.
